# MXene-Embedded Electrospun Polymeric Nanofibers for Biomedical Applications: Recent Advances

**DOI:** 10.3390/mi14071477

**Published:** 2023-07-23

**Authors:** Bishweshwar Pant, Mira Park, Allison A. Kim

**Affiliations:** 1 Carbon Composite Energy Nanomaterials Research Center, Woosuk University, Wanju 55338, Republic of Korea; bisup@woosuk.ac.kr; 2 Woosuk Institute of Smart Convergence Life Care (WSCLC), Woosuk University, Wanju 55338, Republic of Korea; 3 Department of Automotive Engineering, Woosuk University, Wanju 55338, Republic of Korea; 4 Department of Healthcare Management, Woosong University, Daejon 34606, Republic of Korea

**Keywords:** MXene, polymer, electrospinning, nanofibers, biomedical applications

## Abstract

Recently MXenes has gained immense attention as a new and exciting class of two-dimensional material. Due to their unique layered microstructure, the presence of various functional groups at the surface, earth abundance, and attractive electrical, optical, and thermal properties, MXenes are considered promising candidates for various applications such as energy, environmental, and biomedical. The ease of dispersibility and metallic conductivity of MXene render them promising candidates for use as fillers in polymer nanocomposites. MXene–polymer nanocomposites simultaneously benefit from the attractive properties of MXenes and the flexibility and facile processability of polymers. However, the potentiality of MXene to modify the electrospun nanofibers has been less studied. Understanding the interactions between polymeric nanofibers and MXenes is important to widen their role in biomedical applications. This review explores diverse methods of MXene synthesis, discusses our current knowledge of the various biological characteristics of MXene, and the synthesis of MXene incorporated polymeric nanofibers and their utilization in biomedical applications. The information discussed in this review serves to guide the future development and application of MXene–polymer nanofibers in biomedical fields.

## 1. Introduction

**Figure 1:**
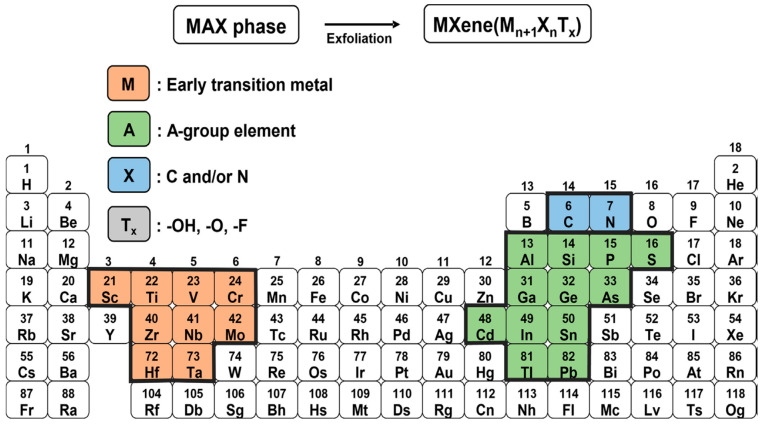
Periodic table showing the composition of the MAX phase and MXene. [5] Reprinted with the permission from Journal of Industrial and Engineering Chemistry © 2022 The Korean Society of Industrial and Engineering Chemistry. Elsevier B.V.

MXenes are the recently emerged member of multifaceted two-dimensional (2D) transitional metal carbides and nitrides, which are derived from MAX phases, where M represents layers of transition metals, A represents A-group element (mostly IIIA and IVA), and X represent either carbon or nitrogen (Figure 1). They are represented with the universal formula M_n_ + 1X_n_, where n = 1, 2, or 3. MXenes were first invented by Professors Yury Gogotsi and Michel W. Barsoum with their group at Drexel University in 2011 [1]. In general, MXenes possess several outstanding features such as high electrical conductivity, good mechanical stability, large surface area, rich surface chemistry, hydrophilicity, ease of surface functionalization, superior near-infrared (NIR) responsiveness, biocompatibility, and excellent optical properties [2]. Due to these interesting features, MXenes and their derivatives have been widely applied in various fields such as energy storage, photocatalysis, sensor, drug delivery, tissue engineering, EMI shielding, etc. [2,3,4].

## 2. Synthesis of MXenes

**Figure 2:**
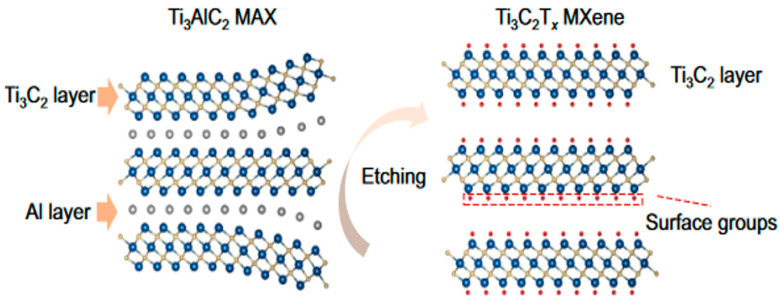
Schematic showing the etching of Ti3 AlC2 MAX phase precursor into Ti3 C2 Tx MXene. [24] Adopted with the permission from Trends in Chemistry © 2020 Elsevier Inc.

Typically, MXenes are synthesized via the selective removal of the “A” layer from their MAX phase by etching. Due to the stronger chemical bonds between the Mn + 1Xn layer and the “A” element layer of MAX phases, it is difficult to prepare MXenes by mechanical exfoliation. However, various mix bonds in the M-A layer (ionic, covalent, and metallic) are weaker than the metallic bonds of the M-X layer. This feature enables the possibility of selective removal of the A layer from the parent phase by etching. In 2011, Gogotsi, Barsoum, and colleagues first synthesized titanium carbide (Ti_3_C_2_) by chemically etching MAX phases of Ti_3_C_2_ using HF [1]. Since then, various etching methods have been proposed as alternative acidic solutions such as the mixture of hydrochloric acid with lithium fluoride [6], hydrochloric acid with sodium fluoride [7], ammonium hydrogen fluoride [8], and ammonium hydrogen fluoride in organic polar solvents [9]. Additionally, several top-down and bottom-up methods have been explored for the synthesis of MXenes. These methods include urea glass method [10], chemical vapor deposition [11], molten salt etching [12], hydrothermal fabrication [13], and electrochemical preparation [14]. Table 1 shows various techniques for the synthesis of MXenes and their applications. Among them, chemical vapor deposition and wet etching techniques have been widely reported for the synthesis of MXenes [4,15,16]. A schematic diagram showing the etching of the Ti_3_AlC_2_ MAX phase into the Ti_3_C_2_T_x_ MXene is given in Figure 2. Table 2 depicts the commonly used method for MXenes synthesis and summarizes their advantages and disadvantages.

**Table 1:** Various techniques for the synthesis of MXenes and their applications [17].

Material	Method of Synthesis	Applications	Refs.
2D TiVC solid solution	Hydrothermal	Raman scattering substrate	[18]
Oxygen-rich Ti_2_C	Tetramethylammonium hydroxide etching	sensor	[19]
Ti_3_AlC_2_	Molten Salt-Shielded Synthesis	Lithium-ion storage	[20]
Ti_3_C_2_T_x_	In situ	Enhanced Optical properties	[21]
Ti_2_AlC	Electrochemical etching	Synthesis of MXene	[14]
Ti_3_AlC_2_	Molten salt approach	Water splitting	[22]
Ti_3_AlC_2_	Room temperature etching with halogens	Synthesis	[23]

## 3. Properties of MXenes

MXenes are materials of interest due to their outstanding physicochemical properties. These properties make the MXenes suitable for various applications. The unique properties of MXenes, such as physical, chemical, magnetic, thermal, electrical, and mechanical, can be controlled by the MAX phase, etching process, and surface functional groups. Therefore, the MXenes properties can be modified on the basis of their applications.

### 3.1. Electronic Properties

Similar to the MAX phase, the bare MXenes are metallic. However, the surface functionalization (OH, F, O termination) can make them semiconducting [34]. The electrical properties of MXenes are affected by several factors such as preparation process, etching, surface groups, elemental composition, and some surrounding conditions, including humidity, temperature, pH, etc. [35,36]. The electric properties are mostly exploited in the energy storage field. However, they can also be applicable in biomedical areas such as the detection of biomolecules, health detection systems, and so on [37].

### 3.2. Mechanical Properties

The mechanical properties of MXenes are closer to various 2D materials (graphene, boronnitride, and molybdenum disulfide). It has been found that various factors such as the atomic layer thickness, porosity, interlayer spacing, the method of preparation, surface terminations, and composition influence the mechanical properties of the MXenes [38,39]. The mechanical properties depend on the surface termination in the order of O, F, and OH [40]. The mechanical properties of MXenes are important for biomedical applications, especially if the material is going to be used for implants [41].

### 3.3. Magnetic Properties

As MXenes can be composed of a wide variety of transition metals, MXenes can possess magnetic properties. Most of the reported magnetic MXenes are based on magnetic transition metals [42]. In addition, defects in monolayers and surface termination also bring magnetic properties in MXenes [38,43]. The magnetic properties of MXenes have been exploited in spintronic devices, electromagnetic interface shielding, and data storage applications.

### 3.4. Thermal Properties

So far, thermal conductivity of only Ti_3_C_2_T_x_ has been evaluated. Simulation studies have shown low thermal expansion coefficients and higher thermal conductivities of MXenes compared to that of the phosphorene and MoS_2_ monolayer [38,44].

### 3.5. Optical Properties

MXenes show strong plasmonic resonance, broad optical transparency window, nonlinear optical performance, transparency, photothermal conversion, etc. It should be noted that these properties are also affected by the functional groups. For example, fluorinated and hydroxyl terminations show similar characteristics, contrasting with oxygen ones. In the visible range, -F and -OH terminations reduce absorption and reflectivity, whereas, in the UV region, all terminations enhance the reflectivity as compared to the pristine MXenes [45]. The MXene’s ability to interact with light can have a significant impact on some biomedical applications such as bioimaging, biosensing, photothermal therapy, etc. Recently, MXene quantum dots (QDs) have aroused widespread interest in biomedical fields due to their enhanced optical properties compared to their counterparts [46].

Table 3 summarizes the various properties of MXenes.

## 4. Biomedical Applications of MXenes

**Figure 3:**
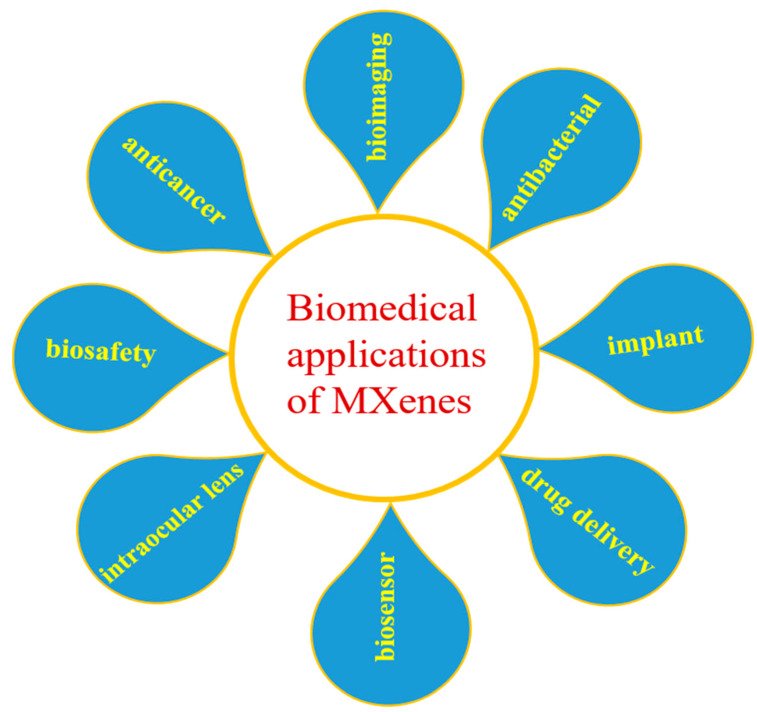
Schematic diagram showing the biomedical applications of MXenes.

In recent years, MXenes have gained tremendous research interest due to their fascinating physiochemical properties (Section 3). These properties make MXene suitable for various fields such as energy storage, catalysis, biomedical, electronics, sensor, solar cells, electromagnetic interference shielding, etc. [2,38,50]. Due to their promising features such as hydrophilicity, biocompatibility, biodegradability, high absorption efficiency over the near-infrared region, and high light-to-heat conversion efficiency, ease of functionalization, MXenes are considered promising materials in biomedical fields. An ideal biomaterial must not be toxic and should be compatible with the physiological environment, be biodegradable, have optimum mechanical properties, and have the ability to overcome biological rejection [51,52]. The intrinsic features of MXenes may not fulfill the requirements in biomedical applications. To overcome this limitation, the integration of MXenes with diverse functional components or fabrication of MXene-based composites have been employed [53,54,55]. In addition, surface modification and functionalization also improve their properties. These strategies are also helpful to extend the applications of MXenes in a wide range. So far, MXenes and their composites have been studied for biosafety, implant, bioimaging, cancer therapy, biosensor, drug delivery, intraocular lenses, antibacterial agents, etc. [38,39,56]. Figure 3 summarizes the biomedical applications of MXenes.

## 5. MXene–Polymer Composites

Preparing composites is considered an effective strategy to develop stable and efficient materials. The composite materials showed improved electronic, magnetic, mechanical, thermal, optical, and structural properties for advanced applications. As discussed earlier (Section 4), several limitations of MXenes can be overcome by forming composites. As a result of two-dimensional (2D) morphology, layered structure, flexibility, and hydrophilicity, MXenes can effectively make a composite with polymers, metal/metal oxides, ceramics, carbons, etc. [57,58]. Among them, the MXene–polymer composites have attracted a great deal of attention in the last decades. MXene–polymer composites can simultaneously exploit the properties of MXenes and the flexibility, processability, and low toxicity of the polymers, leading to many amazing results. The synthesis of MXene–polymer composite was first reported by Ling et al. in 2014 [59]. In this study, the authors mixed Ti_3_AlC_2_ with poly(diallyldimethylammonium chroride) (PDDA) and polyvinyl alcohol (PVA) to produce a composite. Since then, several research groups have prepared MXene–polymer composites for various applications. So far many polymers have been reported, such as polyacrylic acid (PAA) [60], PVA [61,62], polyethylene (PE) [63], polyethylene oxide (PEO) [64], polypropylene (PP) [65], polystyrene (PS) [66], polyimide (PI) [67], acrylamide [68,69], silicone [70], urethanes [71], and epoxy [72], etc. Generally, MXene–polymer composites are prepared by physical mixing, in situ polymerization, melt blending, and electrospinning [4,73,74]. Among them, the electrospinning method has been accepted as a simple and effective operation to prepare MXene sheets incorporated in polymer nanofibers composites [55,75,76]. Therefore, by adopting electrospinning strategy, the outstanding features of nanofibers, along with the properties of MXenes, can be exploited. The following section introduces the electrospinning technique to prepare MXene–polymer composites.

## 6. Electrospinning Technique

Electrospinning is known as a simple, versatile, and cost-effective technique for producing nano-to-microfibers from various polymeric solutions or melts [77]. The electrospinning device consists of four components, namely, a high voltage power supply, a capillary tube, a spinneret/nozzle, and a collector. The working mechanism simply involves electrohydrodynamic process, in which a liquid droplet is electrified to create a jet. After elongation and stretching, the jet generates a single thread of fiber which is deposited onto a collector [51,78]. Recently, electrospinning has gained interest of scientific community due to several outstanding properties such as large surface-to-volume ratio, porosity, flexibility, good mechanical properties, etc. [79]. So far, various morphologies of electrospun nanofibers have been reported such as random [80], porous [81], aligned [82], core-shell [79], helical [83], hollow [84], multilayer [85], and janus [86]. The aforementioned properties make them suitable for various applications such as catalysis [87,88], energy storage [89,90], cosmetics [91], filter media [92], sensors [93], fire retardants [94], environmental remediation [95], fuel cells [96], etc. Electrospun nanofibers resemble the extracellular matrix (ECM) of the human body. In addition, the interconnecting porous morphology facilitates attachments, migration, and proliferation of cells. Therefore, in recent years, a huge number of polymers have been electrospun into nanofiber form for biomedical applications such as wound dressing [97], drug delivery [79], dura mater regeneration [51], implants [98], antibacterial agent [99], biosensors [93,100], etc. Researchers have prepared biologically functional nanofibers by introducing tissue enhancers to enhance biocompatibility [101,102]. The introduction of additional moieties such as graphene [103], carbon nanotubes [104], metal/metal oxide nanoparticles [105], drugs [79,97], biological factors [106,107], etc., into electrospun nanofibers has shown promising results.

### MXenes/Polymeric Nanofibers by Electrospinning

MXenes have gained attention as recently discovered two-dimensional (2D) metal carbides/nitrides [56,108]. Previous studies have shown that the electrospinning technique can be used to embed 2D sheets into nanofibers. The electrospun nanofibers provide sufficient surface for the 2D sheets and hold them within the fibers, whereas the nanofibers can benefit from inorganic filling materials for enhanced performance [85,109,110]. Accordingly, some attempts have been made to incorporate MXene sheets into electrospun nanofibers to widen their applications [55,111,112,113]. In this regard, Mayeberger et al. [76] electrospun poly(acrylic acid) (PAA), poly(ethylene oxide) (PEO), poly(vinyl alcohol) (PVA), and alginate/PEO with delaminated Ti_3_C_2_ for the first time and studied the effect of addition of MXene on the structure and properties of the nanofibers. The authors found that the MXene nano-flakes can be encapsulated into the polymer nanofibers by electrospinning technique. Since then, several research groups have incorporated MXene sheets into polymer nanofibers for various applications [55,114,115,116,117].

## 7. Biomedical Applications of Electrospun MXenes/Polymeric Nanofibers

In recent years, several efforts have been made to incorporate the MXene sheets into polymeric nanofibers for biomedical applications such as antibacterial, biocompatibility, biosensing, wound healing, and bone regeneration, etc. (Table 4). From Table 4, it can be concluded that most of the studies are conducted for biocompatibility and antibacterial evaluations.

### 7.1. Cellular Behaviors and Biocompatibility Evaluation

**Figure 4:**
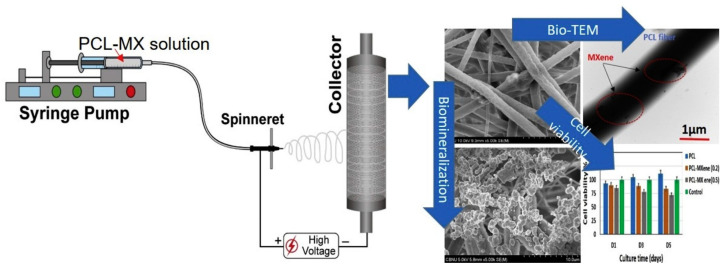
Schematic diagram showing the synthesis of MXene–PCL nanofibers, SEM image of the composite fibers, cell viability, and biomineralization [118]. Reprinted with permission from Colloids and Surfaces A: Physicochemical and Engineering Aspects. © 2019 Elsevier B.V.

Biocompatibility is a prerequisite for any materials for biomedical applications. It should enhance tissue regeneration without inducing any adverse reactions [51]. Awasthi et al. [118] synthesized polycaprolactone (PCL)-Ti_3_C_2_ MXene composite nanofibers by electrospinning for the first time and studied their physiochemical and biological features. The authors prepared Ti_3_C_2_ MXenes by hydrofluoric acid (HF) etching, dispersed them into PCL solution, and finally electrospun the solution into MXene–PCL composite nanofibers (Figure 4). They studied the biocompatibility of the PCL/Mxene nanofibers by using fibroblasts (NIH-3T3) and preosteoblasts (MC3T3-E1) cell lines and found about 70% and 72% of cell viability, respectively, after 5 days of culture. The biomineralization test revealed a successful deposition of calcium phosphate minerals which was due to the better wettability of MXenes. The finding from this study shows the possibilities of utilizing MXene–PCL nanofibers in bone implants. Similarly, Kyrylenko et al. [120] investigated the possibilities of utilizing MXene-loaded polylactic acid (PLA) electrospun nanofibers for nerve guide conduits. The introduction of MXenes not only increased the electrical conductivity of the nanofibers but also showed high biocompatibility. In addition, the composite materials tended to inhibit bacterial adhesion. These all features verified the potentiality of the as-prepared PLA-MXene membranes in creating nerve guide conduits and other biomedical applications. Nan et al. [126] exploited Ti_3_C_2_T_x_ MXene-coated PCL nanofiber conduits for enhancing neurite regeneration and angiogenesis. The in vitro evaluation on nerve regeneration revealed good biocompatibility. They further made a sciatic nerve defect model of SD rats and implanted their composite nanofibers. The authors observed excellent performance by their synthesized materials in promoting nerve regeneration in a long rate sciatic nerve defect. Mayerberger et al. [119] determined the biocompatibility of Ti_3_C_2_T_z_ incorporated chitosan nanofibers by performing an in vitro cytotoxicity test using HeLa cells for 72 h. Their study demonstrated >85% average cell viability at all test concentrations, indicating that the nanofibers were not cytotoxic to HeLa cells. The biocompatibility of the MXene-loaded electrospun nanofibers was also evaluated by Huang et al. [111]. They prepared Ti_3_C_2_ nanosheet-embedded PLLA-PHA composite nanofibers by electrospinning and studied their performance in osteogenesis differentiation on bone marrow-derived mesenchymal stem cells (BMSCs). The MXene composite nanofibers revealed good biocompatibility and enhanced cellular activities. MXene–polymeric nanofiber membranes are also effective in promoting spontaneous osteogenic differentiation [133]. Recently, Lee et al. [124] synthesized MXene NPs-integrated poly(L-lactide-co-ε-caprolactone, PLCL) and collagen (Col) (i.e., PLCL/Col/MXene) nanofibers and evaluated the spontaneous osteogenic differentiation of MC3T3-E1 preosteoblasts. The PLCL/Col/MXene nanofiber resembled the structure of the natural extracellular membrane (ECM) and possessed excellent physiochemical properties, thereby providing a favorable microenvironment for unprecedented cellular behavior of MC3T3-E1 preosteoblasts.

### 7.2. Antibacterial Activities

**Figure 5:**
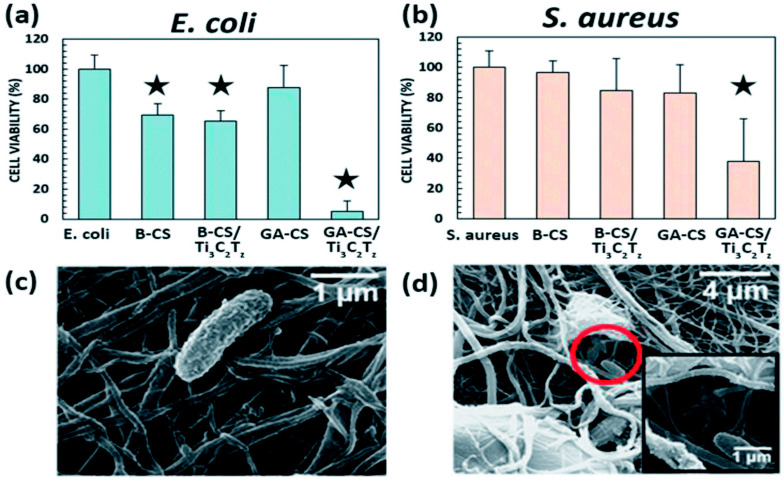
Antibacterial activity against *E. coli* (**a**) and *S. aureus* (**b**). SEM images showing intact (**c**) and destroyed (**d**) *E. coli* on Ti_3_C_2_T_z_/CS nanofibers [119].

Implant-related infection is a serious issue in the healthcare system which may lead to acute/chronic inflammation and foreign body reaction resulting in microbial colonization and infection. Developing antibacterial biomaterial is a promising strategy to prevent implant-related infections. Several studies have shown that the antibacterial property can be induced into the electrospun polymeric nanofibers by loading the antibacterial agents [92,97,134,135]. Rasool et al. [136] introduced Ti_3_C_2_Tx MXenes as a new family of 2D antibacterial materials for the first time. Recently, some efforts have been made to synthesize antibacterial polymeric nanofibers by incorporating MXene sheets via an electrospinning technique [119,129,132]. In this regard, Mayerberger et al. [119] incorporated Ti3C2Tz flakes into chitosan (CS) nanofibers by electrospinning and tested their antibacterial performance against Escherichia coli (*E. coli*) and Staphylococcus aureus (*S. aureus*) bacteria (Figure 5). After 4 h of treatment, the CS nanofibers containing 0.75 wt % of MXene exhibited 95% and 62% reduction in colony-forming units on the *E. coli* and *S. aureus*, respectively. According to the authors, the antibacterial property was attributed to the direct mechanical destruction by MXene penetration through bacterial membranes. Wu et al. [129] prepared graphene oxide (GO)/MXene-loaded 3-hydroxybutyrate-co-hydroxyvalerate (PHBV) fibers by electrospinning. The antibacterial rate of the composite fibers was higher than 95% against both *E. coli* and *S. aureus*. The antibacterial nature of the prepared composite fibers was mainly attributed to the Mxene. Wang et al. [132] developed an antibacterial nanofiber membrane composed of polyvinylidene fluoride/Bi_4_Ti_3_O_12_/Ti_3_C_2_T_x_ (PVDF/BTO/Ti_3_C_2_T_x_) by an electrostatic spinning process. The composite nanofibers showed excellent antibacterial activity (higher than 99%) against *E. coli* and *S. aureus*. According to the authors, the excellent antibacterial activity could be due to the combined effect of reactive oxygen species (ROS) and hyperthermia induced by light irradiation.

### 7.3. Wound Healing

**Figure 6:**
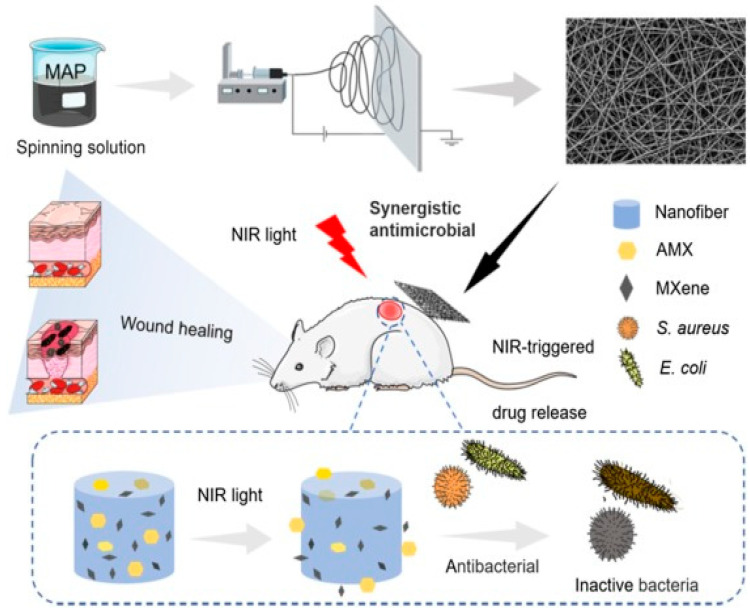
Schematic diagram showing the preparation of MXene–AMX–PVA nanofiber membrane and their antibacterial and wound healing applications [122]. Reprinted with permission from Colloids and Surfaces. B: Biointerfaces. © 2021 Elsevier B.V.

In recent years, electrospun nanofiber membranes have been extensively investigated for wound-healing applications. Electrospun nanofibers can be designed in two-dimensional (2D) and three-dimensional (3D) configurations [137] which closely emulate the tensile strength and elastic modulus of human skin [138,139]. Previous studies have demonstrated that incorporating 2D nanosheets into electrospun nanofibers enhances the mechanical properties of membranes and improves biocompatibility, which makes them suitable for wound dressing and scaffold applications [140,141,142,143]. Many researchers have investigated the significance of MXenes in wound dressing applications [144,145]. Among many elements, titanium carbide (Ti_2_C_2_Tx) has been used mainly for wound healing due to the antimicrobial and biocompatible nature of titanium [145]. However, the direct use of MXene sheets in surgical incisions may create some problems. For example, MXene nanosheets may be shed, and direct contact with tissue could potentially result in MXene nanosheets persisting in tissue that cannot be removed [128]. Loading MXene sheets into nanofibers could be helpful to avoid direct contact between MXene nanosheets and tissue. In this regard, Xu et al. [122] prepared amoxicillin and MXene-loaded PVA nanofiber membrane as a biocompatible antibacterial and wound dressing material. The author studied the antibacterial properties and in vitro and in vivo biocompatibility of the composite membrane (Figure 6). This study showed that under the lower power density NIR irradiation, MXene generated hyperthermia, which inhibited the bacterial growth and accelerated MAX release. The authors reported up to 96.1% and 99.1% of bacterial inhibition rates in the case of *E. coli* and *S. aureus*, respectively. The wound healing was tested on mice model and the results showed that the nanofiber membrane was effective in enhancing the wound healing rate upon laser irradiation. Diedkova et al. [130] prepared several layers of Ti_3_C_2_T_x_ MXene immobilized polycaprolactone (PCL) nanofibers and studied their structural, chemical, electrical, and biological properties. It was observed that the double and triple coatings on PCL fibers showed good performance for attachment and proliferation with antibacterial properties. The obtained results from this study demonstrated the potentiality of the MXene–PCL nanofibers for tissue engineering.

### 7.4. Drug Release

Achieving effective and fast wound healing is a great challenge. Recently, drug- or biomolecule-incorporated nanofibers have been explored for rapid wound healing and other biomedical applications [79,101,146,147]. However, such nanofibers suffer from the burst release of the active components and cannot be used for the longer term [79]. In this regard, Jin and coworkers [123] developed near-infrared (NIR) and temperature-responsive MXene nanobelt fibers loaded with vitamin E with controlled release ability and used them for wound healing applications. They prepared composite nanofibers composed of polyacrilonitrile and polyvinypyrrolidone along with MXenes. Additionally, a heat-sensitive layer P(AAm-co-AN-CO-Vim) copolymer (PAAV) was added to control the release rate by temperature regulation. The developed nanofibers showed suitable wetting and spreading effects, which allow easy contact with the surface of the skin to exert its photothermal properties. According to the authors, when NIR light (1.0 W) was applied, the temperature rapidly rose to 60 °C in 2 min. The highest temperature was recorded at 65 °C. The temperature-responsive fiber composite demonstrated good biocompatibility. Most importantly, the MXenes helped in releasing the antibiotics, thereby enhancing the antibacterial activity. The long-term release of vitamin E was achieved, which helped in wound healing.

### 7.5. Wearable Electronics for Health Monitoring

The development of wearable electronics has become an established issue in the healthcare system. Some of the exciting features of the MXenes such as large surface area, electrical conductivity, excellent piezoresistive behavior, and solution processibility make them suitable candidates for wearable sensing applications [125,148,149]. In order to obtain high mechanical strength, MXene–polymer composites have been prepared and there is still an ongoing need to obtain more sensitive and flexible pressure sensors [150,151]. Recently, Sharma et al. [121] prepared MXene (Ti_3_C_2_T_x_)/poly(vinylidene fluoride trifluoro ethylene) (PVDF-TrFE) composite nanofibers sandwiched in between biocompatible poly-(3,4-ethylenedioxythioiphene)polystyrene sulfonate/polydimethylsiloxane electrodes and exploited them as a wearable capacitive pressure sensor. It was observed that the sensitivity of the nanofiber-based sensor was enhanced by MXene loading. According to the authors, the sensor can be used to determine the health condition of patients by inspecting various physiological signals such as pulse rate, respiration, movement of muscle, eye twitching, etc. Similarly, Leong et al. [125] prepared an MXene-based strain sensor composite strain sensor by depositing Ti_3_C_2_T_x_ doped polypyrrole on flexible electrospun PVDF nanofibers. Thus, prepared electrospun composite fibers provide a percolation for conductive filler network formation, which is the key factor of excellent sensitivity. The sensor can be adapted for real-time human motion detection. Cui et al. [127] demonstrated a multifunctional and breathable MXene–Polyurethana mesh (MPM) e-skins for long-term health monitoring. Embedding the MXene nanoplates into the PU nanomesh network brought several outstanding features such as high breathability, good mechanical stability, super sensitivity, and excellent durability. According to the authors, the synthesized MPM can monitor multiple physiological signals such as pulse, respiration, voice recognition, and joint movement. For example, when the MPM was used as electrocardiograph (ECG) electrodes, the electrode–skin contact impedance, signal-to-noise ratio, and breathability were recorded as 4.68 kΩ at 1 kHz, 16.5 dB, and 2.1338 kg/m^2^/day, respectively. Furthermore, the study showed that the as-designed e-skins can be continuously used for at least seven days.

## 8. Conclusions

Recently, the incorporation of MXene into various polymeric nanofibers has been prepared for various applications. This review discussed the synthesis of MXene–polymeric nanofibers membranes by electrospinning technique and their biomedical applications. The mechanical, physiological, and biological properties of the electrospun nanofiber membranes are important characteristics for biomedical applications. Designing composite nanofibers with MXene sheets further enhanced these properties, widening their applications in biomedical fields. The MXene–polymer nanofibers composite has shown promising candidacy in antibacterial, wound healing, cellular differentiation, bone tissue regeneration, neural tissue guidance, and health monitoring systems. However, the toxic and corrosive liquids used for the synthesis of MXene may bring undesirable impacts. In addition, it is challenging to disperse MXene sheets into polymeric solution and prepare nanofibers with well-distributed MXene sheets. It should be noted that most of the studies have used titanium carbide as a filler material in polymer nanofibers, whereas the other MXenes are yet to be explored in electrospinning systems for biomedical applications. The practical applications are another issue. So far, there are very few reports on such nanofiber composites, most of the studies are at the lab scales, and further investigations are needed for the clinical applications. A comprehensive understanding of various MXenes and polymer nanofiber forming by electrospinning is essential to address the above issues. Therefore, further research works are needed to overcome the aforementioned problems.

## Figures and Tables

**Table 2:** Common methods for MXenes synthesis and their advantages and disadvantages [25].

S.N.	Method	Advantages	Disadvantages	Refs.
1.	HF etching	- Suitable for the wide range of MXene compounds. - Allows simultaneous intercalation during etching.	- Hazardous. - It creates –F terminated MXenes, which have a detrimental impact on applications. - Additional cleaning process is required.	[26,27]
2.	Alkali etching	- Feasible to remove the –F terminals from MXenes. - Safe.	- Harsh reaction conditions may be required. - Mechanism of the organic base reaction is unclear.	[28,29]
3.	Electrochemical etching	- Control over the surface terminations - safe due to fluorine-free synthesis.	- Expensive set up required.	[30]
4.	Molten salt etching	- Lewis acidic salts are efficient to prepare MXenes from MAX phase, including Zn, Ga, and Si.	- Requires harsh experimental conditions- - Produce –F terminated MXenes.	[31]
5.	Plasma-enhanced pulsed-laser deposition	- Crystal structure can be controlled.	- Energy intensive procedure - Unclear working mechanism.	[32]
6.	Template-assisted method	- Surface terminations can be controlled.	- Limited template. - Energy-intensive process.	[33]

**Table 3:** Properties of MXenes [38,47,48].

S.N	Properties	Remarks	Refs.
1	Electronic and electric	- MXenes possess outstanding electrical conductivity. - These properties can be influenced by several factors such as source of MAX, synthesis condition, and surface functionalization, etc.	[38]
2	Mechanical	- Depending on the surface terminations in the order of O, F, and OH.	[40]
3	Magnetic	Magnetic features can be adjusted by surface functionalization. Some are ferromagnetic. Example: Ti_2_N, Cr_2_C, and Ti_2_C - some are anti-ferromagnetic. Example: Cr_2_N and Mn_2_C	[38]
4	Thermal	Simulation studies predicted low thermal expansion coefficients and higher thermal conductivities than phosphorene and MoS_2_ monolayer.	[44,49]
5	Optical	MXenes show strong plasmonic resonance, broad optical transparency window, nonlinear optical performance, transparency, photothermal conversion, etc. - the optical properties are also affected by the functional groups.	[45]

**Table 4:** MXene–polymer nanofibers fabricated by electrospinning process for biomedical applications.

Polymer	MXene	Applications	Ref.
Polycaprolactone (PCL)	Ti_3_AlC_2_	Biocompatibility evaluation	[118]
Chitosan	Ti_3_C_2_T_z_	Antibacterial medium	[119]
Polylactic acid (PLA)	Ti_3_C_2_T_x_	Antibacterial and biocompatibility evaluation	[120]
poly(vinylidene fluoride-trifluoroethylene) (PVDF-TrFE)	Ti_3_C_2_T*_x_*	Sensor to determine the health condition of patients	[121]
PVA	Ti_3_C_2_	Treatment of wound infection	[122]
PLLA-PHA	Ti_3_C_2_	Tissue engineering	[111]
polyvinylpyrrolidone (PVP)-PAN	Ti_3_C_2_	Wound healing	[123]
PLCL/collagen	Ti_3_AlC_2_	Bone tissue regeneration	[124]
PVDF	Ti_3_C_2_T_x_	Sensor for body movement detection	[125]
PCL	Ti_3_C_2_T_x_	NeuriteRegeneration and Angiogenesis	[126]
PU	Ti_3_C_2_	ECG monitoring system	[127]
PLA, gelatine	Ti_3_C_2_T_x_	- Inhibition of tumor reoccurrence - Wound healing	[128]
PLA	Ti_3_C_2_T_x_	Good biocompatibility, Inhibition of bacterial adhesion Applicable in the development of neural guidance conduit	[120]
PHBV	Ti_3_C_2_T_x_	Antibacterial activities	[129]
PCL)	Ti_3_C_2_T*_x_*	Tissue engineering scaffolds	[130]
PVDF	Ti_3_C_2_T*_x_*	Bone Regeneration	[131]
PVDF	Ti_3_C_2_T*_x_*	Light-responsive antibacterial material	[132]
